# Epidemiology of a *Daphnia-*Multiparasite System and Its Implications for the Red Queen

**DOI:** 10.1371/journal.pone.0039564

**Published:** 2012-06-25

**Authors:** Stuart K. J. R. Auld, Spencer R. Hall, Meghan A. Duffy

**Affiliations:** 1 School of Biology, Georgia Institute of Technology, Atlanta, Georgia, United States of America; 2 Department of Biology, Indiana University, Bloomington, Indiana, United States of America; University of Helsinki, Finland

## Abstract

The Red Queen hypothesis can explain the maintenance of host and parasite diversity. However, the Red Queen requires genetic specificity for infection risk (i.e., that infection depends on the exact combination of host and parasite genotypes) and strongly virulent effects of infection on host fitness. A European crustacean (*Daphnia magna*) – bacterium (*Pasteuria ramosa*) system typifies such specificity and high virulence. We studied the North American host *Daphnia dentifera* and its natural parasite *Pasteuria ramosa*, and also found strong genetic specificity for infection success and high virulence. These results suggest that *Pasteuria* could promote Red Queen dynamics with *D. dentifera* populations as well. However, the Red Queen might be undermined in this system by selection from a more common yeast parasite (*Metschnikowia bicuspidata*). Resistance to the yeast did not correlate with resistance to *Pasteuria* among host genotypes, suggesting that selection by *Metschnikowia* should proceed relatively independently of selection by *Pasteuria*.

## Introduction

Genetic specificity between hosts and their parasites shapes the ecology and evolution of infectious disease [1], [2]. Specificity occurs in numerous host-parasite systems, and arises when the exact pairing of host and parasite genotypes determines the success of infection (e.g. [3], [4], [5], [6], [7]). Genetic specificity has the potential to influence disease phenomena in at least two ways. First, it decreases the likelihood of invasion of parasites into genetically diverse host populations (i.e., the parasite will have a lower reproductive ratio *R*_0_ when all else is equal, [8]) because much of the host population resists infection. Indeed, genetically diverse populations often experience smaller disease epidemics [9], [10], [11], [12], [13]. Second, specificity means that each parasite genotype only selects against a subset of host genotypes (and vice-versa). Therefore, specificity can drive Red Queen coevolutionary dynamics, where the frequencies of host and parasite genotypes cycle through time [14], [15]. Such coevolutionary cycling can maintain genetic diversity in both host and parasite populations [15], [16].

The European freshwater crustacean *Daphnia magna* and its sterilizing bacterial parasite *Pasteuria ramosa* exemplify genetic specificity [5], [7]. Here, specificity stems from a mechanism at the point of entry of *Pasteuria* – the physical attachment of spores to the host oesophageal wall [17], not from a systemic immune response [18], . If infection is successful, *Pasteuria* inflicts high virulence on infected hosts through sterilization [20], [21]. Further, *Pasteuria* epidemics can become large [22], [23], [24], promoting rapid evolution in host populations [23]. In theory, such a combination of genetic specificity, high parasite virulence, and large epidemics promote Red Queen oscillatory dynamics [25], [26], [27]. As predicted by this theory, Red Queen dynamics emerge in the *D. magna-Pasteuria* system [28].

These results suggest that *Pasteuria* could maintain genetic diversity in other susceptible host species. Therefore, *Pasteuria* could act as a general catalyst of Red Queen-driven coevolution in freshwater systems. To test this possibility, we looked for genetic specificity for infection with *Pasteuria* in a natural North American host, *Daphnia dentifera,* using an established experimental design [7]. We also tested for evidence of high virulence in infected hosts, with particular focus on fecundity (since *Pasteuria* castrates its European *D. magna* hosts: [20]). If *Pasteuria*'*s* specificity and virulence effects are general, the European *Daphnia – Pasteuria* story may apply more broadly.

However, Red Queen dynamics could be disrupted by the presence of another, more dominant parasite species. For example, directional selection from a non-specific parasite may shape evolution in the host population more than negative frequency-dependent selection from a less prevalent parasite that exhibits genetic specificity with the host. This consideration is especially important in natural systems where most hosts are susceptible to multiple parasite species [29], [30]. In *D. dentifera* populations, epidemics of the yeast *Metschnikowia bicuspidata* are usually larger and more frequent than *Pasteuria* epidemics [31], [32]. *Metschnikowia* does not, however, exhibit genetic specificity with *D. dentifera* [33]; instead, variation in infection risk stems mainly from variation in host exposure to the parasite [34]. Since the infection genetics differ between the two parasites, a host that resists *Pasteuria* may not necessarily resist *Metschnikowia*. Such parasite-specific resistance (sensu [35]) may disrupt or prevent Red Queen dynamics. To examine this possibility, we compared mean resistance of host genotypes to both parasites.

## Materials and Methods

### Study system

*Daphnia dentifera* is a cyclically parthenogenetic freshwater crustacean widespread in stratified North American lakes [36]. It hosts numerous parasites [32], [37], two of which receive focus here: the bacterium *Pasteuria ramosa* and the yeast *Metschnikowia bicuspidata.* For both parasites, infection occurs when the host encounters transmission spores when filter-feeding [17], [38]. With *Pasteuria*, infection also critically depends on attachment of cells to the oesophagus of the host; this step depends strongly on genotype in the *Daphnia magna* system [17]. In both species, spore release follows death of the host (i.e., both are obligate killers: [39], [40]), however, the two parasites vary in terms of their effects on fecundity and survivorship. Although not previously studied in *D. dentifera*, *Pasteuria* completely sterilizes *D. magna* [20]. In contrast, infection of both *D. magna* and *D. dentifera* by the yeast causes a smaller reduction in fecundity but sharply reduces survival of hosts [40], [41], [42]. In *D. magna*, *Metschnikowia* has a larger impact on host survival than does *Pasteuria* [39].

### Experimental design

The experiments used host and parasite cultures and previously established laboratory protocols. The six clonal genotypes of *Daphnia* (named H1, H4, H9, H29, H37 and H119) and five *Pasteuria* isolates (named A, B, C, D, and G) all originated from Midland Lake, Greene County, Indiana, USA. The five *Pasteuria* isolates were taken from five different infected hosts in 2010 (one isolate per host individual) and were propagated by feeding homogenized, infected hosts to a single host genotype (H119). These isolates likely consist of a mix of *Pasteuria* clones and represent ecologically relevant sub-populations of *Pasteuria* (the same as [7]). The *Metschnikowia* isolate originated from multiple infected hosts collected from Baker Lake, Barry County, Michigan, USA in 2003 and was propagated in a similar manner (on a single host genotype, the “Standard” genotype, also collected in Michigan). We used only this lab-based culture because *Metschnikowia* isolated from different lakes and in different years show no genetic variation in infectivity or virulence on *D. dentifera* [33]. No specific permits were required for the collection of *D. dentifera* (which is not a protected or endangered species), but permission was obtained for access to lakes.

The experimental design involved a factorial manipulation of these hosts and parasites. We crossed the six host genotypes with seven parasite treatments (five *Pasteuria* isolates, one *Metschnikowia* isolate, and a sham-exposure control), replicated 15 times, for a total of 630 experimental units. Four of the *Pasteuria* replicates were lost due to accidental death after treatment exposure. Maternal lines consisted of individual *Daphnia* kept under favourable conditions (20°C and 16∶8 hour light/dark) in 40 mL of medium (50% Artificial *Daphnia* medium [43] and 50% filtered lake water); they received ample food (1×10^6^ cells of the alga *Ankistrodesmus falcatus* daily). Experimental animals consisted of second clutch offspring of third-generation maternal lines and were also kept in 40 mL of medium and fed ample food. In the parasite exposure treatments, each neonate (<24 hour old) received a high dose of spores (*Pasteuria*: 2000 spores/mL; *Metschnikowia*: 500 spores/mL) produced by gently crushing and diluting infected hosts. These doses aimed to yield similar infection levels among parasite species (based on pilot data). Sham controls received 100 μL of a *Daphnia* solution (50 uninfected hosts ground in 10 ml nanopure water). Replicates were fed lower food (0.5×10^6^ *Ankistrodesmus* algal cells) during exposure to increase spore uptake by hosts [38]. Treatment exposure lasted 5 days. After the exposure, each host was changed into fresh medium and given ample food (1×10^6^ *Ankistrodesmus* algal cells) again. Hosts were checked daily for reproduction and mortality for 25 days. Medium was refreshed every 2–3 days.

### Data analysis

All analyses were performed using the statistical package R (http://www.r-project.org). We analysed infection risk (that is, the proportion of infected hosts) by testing the fixed effects of host genotype and parasite species using data from only parasite*-*exposed hosts (not sham-exposed hosts). We tested for genetic specificity of *Pasteuria* infection using the fixed effects of host genotype and parasite isolate. Both Generalized Linear Models (GLM) were estimated with a binomial error distribution, and significance of each treatment was evaluated with deviance tests (i.e., a comparison of full model versus the reduced model). However, in previous *Daphnia*-parasite studies, genotype is fitted as a fixed effect, a random effect or both (e.g. [33], [44], [45], ). Therefore, we also fitted GLMs with host and parasite genotype identity as random effects. In all cases, we obtained the same qualitative results. We also tested for a relationship between risk of infection from *Metschnikowia* and *Pasteuria* (averaged over the five isolates) for each host genotype using a Spearman rank correlation.

We examined the fitness consequences of infection by the two parasites using three metrics: production of host offspring (fecundity), host survival, and the instantaneous rate of host population growth, *r* (a composite measure of host fitness calculated using the Euler-Lotka equation). Fecundity was analysed by testing the fixed effects of infection status (infected or not), host genotype, and parasite species. Both host survival and *r* were analysed by testing the fixed effects of host infection status (infected or not) and parasite species. All of these tests used data from parasite-exposed hosts only; for *r,* the means of data from each infection category and parasite species for each host genotype were used.

*Metschnikowia*'*s* capacity to disrupt Red Queen dynamics between *D. dentifera* and *Pasteuria* should be reduced if the host pays an activation cost of resistance to *Metschnikowia* (i.e., if hosts that successfully resist *Metschnikowia* have a lower fitness than hosts that were not exposed to the parasite.) We looked for evidence of an activation cost of resistance to *Metschnikowia* by comparing host fecundity, survival, and *r* in controls (sham-exposures) with hosts that were exposed to parasites but did not suffer infection (sensu [47], [49]). A Cox's proportional hazards analysis was used to test for activation costs in terms of survival, and Welch's two-sample *t-*tests were used to test for activation costs in terms of fecundity and *r.*

We found relatively low overall levels of infection by *Pasteuria*, as well as strong genotype specificity governing infections. This, combined with the likelihood that our parasite isolates contain mixtures of *Pasteuria* genotypes [5], [48], means we cannot be sure that individuals in the *Pasteuria* exposures who did not become infected where exposed to a parasite spore of an infectious genotype. Thus, we cannot test for activation costs of resistance to *Pasteuria.* In any case, activation costs of resistance to *Pasteuria* are unlikely to be evolutionarily important because *Pasteuria* is so virulent. Since it castrates its hosts, the cost of *Pasteuria* infection is so massive that any cost of resistance would have to be exceptionally high to limit Red Queen dynamics.

Fecundity across treatments was examined using a GLM fit with a quasipoisson error distribution (used due to over-dispersion of the fecundity data), and survival analyses used Cox's proportional hazards. Measures of *r* were squared to normalize their distribution prior to analysis with an ANOVA (with type III SS).

## Results

### Genetic specificity for infection risk

**Figure 1:**
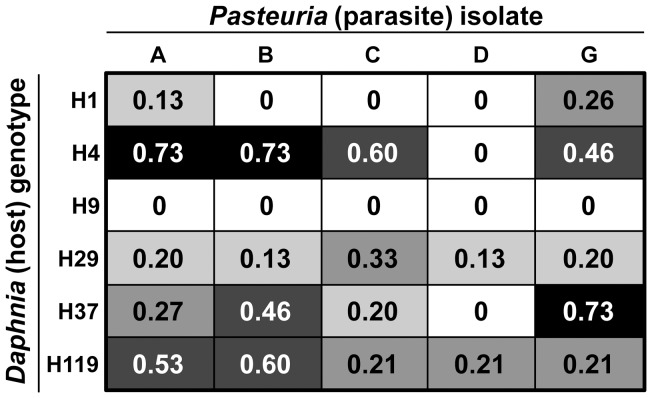
Infection risk for six host genotypes exposed to five isolates of *Pasteuria ramosa* . The matrix shows the proportion of hosts infected for each combination of host genotype (rows) and parasite isolate (columns). Cells show infectivity of the five different *Pasteuria* isolates on each of the six host genotypes. There are five shading categories: 0% infection (white), 1–20%, 21–40%, 41–60%, and 61–100% (black). See Table 1 for statistical details.

**Figure 2:**
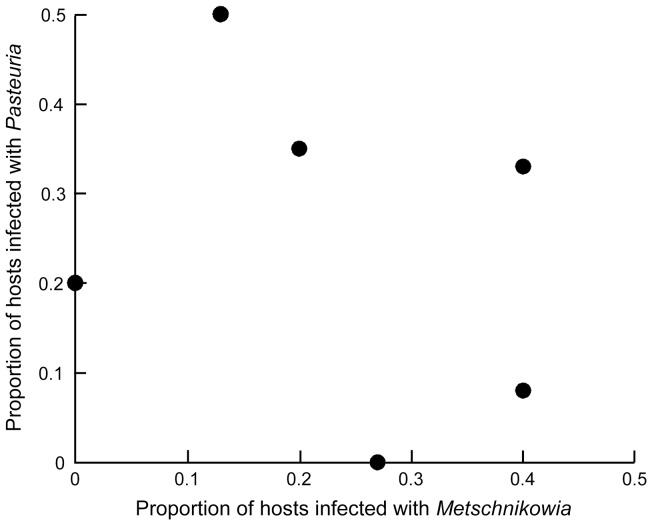
Spearman rank correlation between *Pasteuria* and *Metschnikowia* infectivity. *r*_s_ = −0.38, *p* = 0.46. Each data point is the mean for each host genotype.

Genetic specificity for infection risk arose at both a within-species level (for the bacterium *Pasteuria*) and between parasite species. Infection risk from *Pasteuria* depended on the specific combination of host and parasite genotypes (Figure 1), as evidenced by a host genotype x *Pasteuria* isolate interaction (Table 1). This interaction remained significant when we treated both host genotype and parasite isolate as random effects (χ ^2^ = 15.39, df = 3, *p*<0.01). As expected based on prior studies, host genotype strongly influenced infection risk from *Metschnikowia* (Figure 2; Table 1). Infection risk also depended on an interaction between host genotype and parasite species (Table 1). This interaction remained significant when host genotype was coded as a random effect (χ ^2^ = 22.81, df = 2, *p*<0.0001). There was, however, no correlation between a genotype's risk of infection by *Metschnikowia* and its overall risk of infection by *Pasteuria* (*r*_s_ = −0.38, *p* = 0.46; Figure 2).

**Table 1:** Summary statistics of models testing the effects of host genotype, parasite species and *Pasteuria* isolate on infection risk.

Source	DF	L-R χ ^2^	*p*
Model 1: All parasite-exposed hosts			
Host genotype	5	61.65	**<0.0001**
Parasite species	1	0.13	0.72
Host genotype x Parasite species	5	32.52	**<0.0001**
Model 2: *Pasteuria* only (5 isolates)			
Host genotype	5	92.97	**<0.0001**
*Pasteuria* isolate	4	35.06	**<0.0001**
Host genotype x *Pasteuria* isolate	20	42.47	**<0.01**
Model 3: *Metschnikowia* only (1 isolate)			
Host genotype	5	13.22	**<0.05**

DF: degrees of freedom; L-R χ ^2^: likelihood-ratio χ ^2^ statistic; *p*: p-value of test. Bolded values are significant at alpha  = 0.05 level.

### Parasite virulence

**Figure 3:**
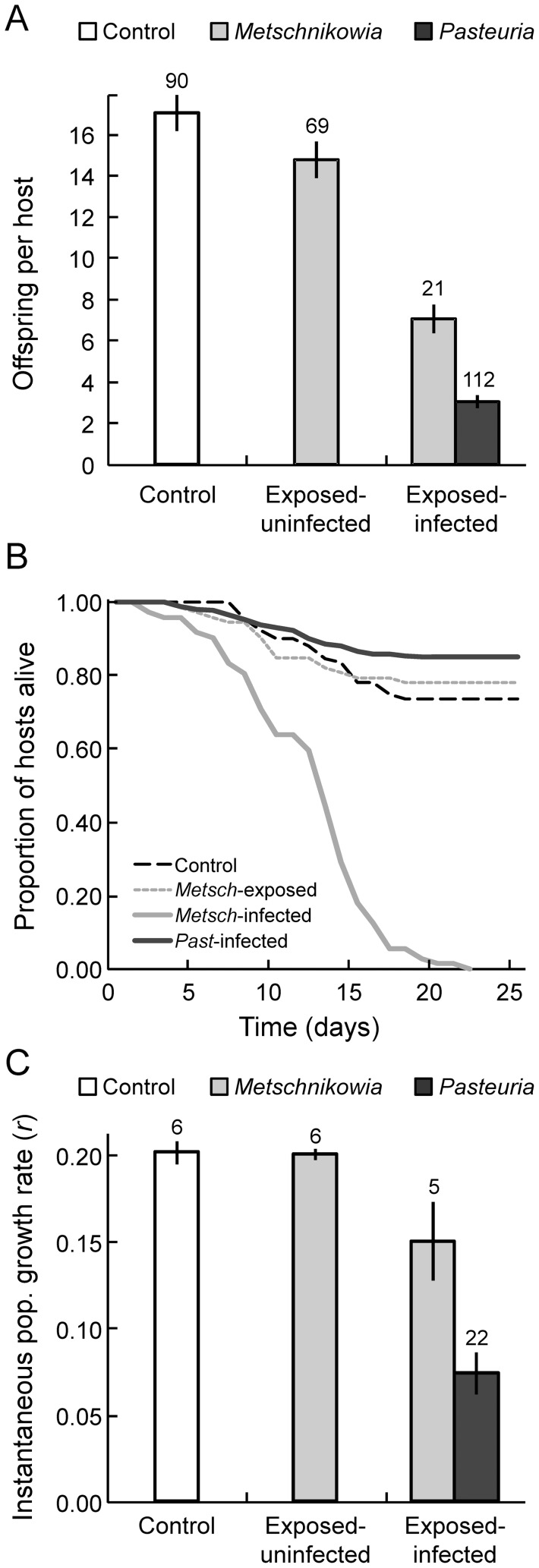
Measures of host fitness in different treatments according to infection status. A: production of host offspring (fecundity) over 25 days; B: survival of hosts; and C: instantaneous rate of population growth of hosts (*r*), a composite measure of host fitness. Error bars denote one standard error. In panel B, “*Metsch*-exposed” indicates animals who were exposed to *Metschnikowia,* but who did not become infected. In all three panels, data were pooled across host genotypes and parasite isolates. Numbers of replicates are shown above bars; for *r,* the means of data for each host genotype from each infection category were used, resulting in lower sample sizes.

Infection with either parasite substantially reduced fecundity in infected hosts (Figure 3A). However, the extent of this virulence on host fecundity differed between the parasites (i.e., there was a significant infection status x parasite species interaction; Table 2): *Pasteuria*-infected hosts suffered a greater reduction in fecundity than those infected with *Metschnikowia* (Figure 3A). The impact of infection on fecundity did not vary across host genotypes (that is, there was no infection status-by-host genotype interaction; Table 2). Host survival also strongly depended on infection status and parasite species: *Metschnikowia-*infected hosts died much earlier than either *Pasteuria-*infected hosts or uninfected hosts (Table 2; Figure 3B). Finally, the instantaneous rate of population growth (*r*; our composite measure of fitness) depended on both infection status and parasite species: infection with either parasite reduced *r*, but *Pasteuria* infections reduced *r* more than *Metschnikowia* infections. Thus, *Pasteuria* was more virulent (Table 2; Figure 3C).

**Table 2:** Summary analysis of fitness components of hosts: fecundity, survival, and instantaneous rate of population growth, *r*.

Host fitness measures	Host fecundity	Host survival	Host *r*
†All parasite-exposed hosts			
Infection status (Inf)	*F*_1,535_ = 298.69***	κ^2^_1_ = 4.94*	*F*_1,59_ = 12.80***
Host genotype (Host geno)	*F*_5,535_ = 2.30*	–	–
Parasite species (Para sp.)	*F*_1,535_ = 0.16	κ^2^_1_ = 9.35**	*F*_1,59_ = 0.67
Inf x Host geno	*F*_5,535_ = 1.71	–	–
Inf x Para sp.	*F*_1,535_ = 12.92***	κ^2^_1_ = 0.81	*F*_1,59_ = 13.00***
Host geno x Para sp.	*F*_5,535_ = 0.91	–	–
Inf x Host geno x Para sp.	*F*_3,535_ = 0.06	–	–
*Pasteuria* only (5 isolates)			
*Pasteuria* isolate	–	–	*F*_4,47_ = 0.95
*Metschnikowia* only (1 isolate)			
Host genotype	–	κ^2^_4_ = 5.46	–

† Data were pooled across host genotypes and parasite isolates Host *r* was analysed using the means for each host genotype. *p<0.05, ** *p*<0.01, *** *p*<0.001.

### Costs of resistance to *Metschnikowia*

Host fecundity did not differ between hosts that were exposed to *Metschnikowia* but not infected and those in our control treatment (control, *n = *90; *Metschnikowia-*exposed, *n = *69; Welch's two-sample *t* = 0.45, df = 110.74, *p = *0.65: Figure 3A); neither host survival (Cox's proportional hazards: χ ^2^ = 0.10, *p = *0.75; Figure 3B) nor the instantaneous rate of host population growth, *r* (Welch's two-sample *t* = 0.14, df = 7.31, *p* = 0.89; Figure 3C) differed between these two groups (that is, *Metschnikowia*-exposed and control). Thus, there was no evidence for a fitness cost of mobilising resistance mechanisms against *Metschnikowia* (Figure 3).

## Discussion

Red Queen dynamics require two essential ingredients. The first is genetic specificity, where infection depends on the precise combination of host and parasite genotypes [15]; the second is high virulence [25], [26], where infection by parasites causes dramatic loss of host fitness. A canonical example of genetic specificity occurs in the European *Daphnia magna-Pasteuria* host-parasite system [5], [7]. We found *Pasteuria* produces a similar pattern of specificity in a North American host, *Daphnia dentifera*: infection risk depended on both host genotype and parasite isolate (Figure 1). Also, just as in *D. magna, Pasteuria* castrates *D. dentifera,* thus greatly depressing fecundity (but not survival: Figure 3; [40], [50]). As a result, fitness is much reduced in *Pasteuria-*infected *D. dentifera* (Figure 3). Therefore, just as in the *D. magna* system, *Pasteuria* in this North American system might also maintain host genetic diversity, become adapted to locally common host genotypes [51], [52], and promote long-term coevolutionary Red Queen dynamics [28].

In addition to *Pasteuria*, *D. dentifera* populations often encounter *Metschnikowia,* a very different parasite. Infection of *D. dentifera* by *Metschnikowia* does not depend on the specific genotype to which it is exposed [33]; instead, genetic variation for host susceptibility stems from host variation in risk of exposure to the parasite [34]. This may explain the absence of activation costs of resistance to *Metschnikowia*: hosts that resisted the parasite may have prevented it from passing the gut wall and stimulating a potentially costly immune response (sensu [18]).

Here, we found that virulence caused by *Metschnikowia* arose mainly through reduced host survival in *D. dentifera* (Figure 3B; similar to *Metschnikowia* infection in *D. magna* hosts: [40]). While *Metschnikowia* killed hosts relatively quickly, it reduced host fecundity much less than *Pasteuria* did (Figure 3A). As a result, *Metschnikowia* reduced per capita host fitness (*r*) to a lesser extent than *Pasteuria* (Figure 3C). Nevertheless, despite the lack of genetic specificity and lower virulence, *Metschnikowia* can still drive evolutionary change of hosts during epidemics. Indeed, large *Metschnikowia* epidemics can substantially reduce host population densities [31] and impose both directional and disruptive selection on host populations [53], [54].

Due to differences in specificity and virulence, these two parasites may exert different ecological impacts on host populations. Theory suggests that specificity can reduce invasion success of parasites; i.e., they will have a lower reproductive ratio *R*_0_ (especially when host populations are genetically diverse: [8]). Indeed genetically diverse host populations of *Daphnia magna* have smaller epidemics of *Pasteuria* [9], [10]. Specificity may thus (partially) explain why *Pasteuria* epidemics occur more rarely and remain much smaller than epidemics of *Metschnikowia* in natural populations of *D. dentifera* [32]. However, even though they are relatively small, *Pasteuria* epidemics may still be ecologically important: theory predicts that, all else being equal, parasites that strongly reduce fecundity (rather than survival) should reduce host population densities, even during small epidemics [55].

Correlations between host resistance to *Pasteuria* and *Metschnikowia* could either amplify or dampen Red Queen oscillations. A negative correlation would indicate antagonistic pleiotropy, where resistance to *Pasteuria* would come at a cost to resistance to *Metschnikowia* [56]; this could bolster Red Queen dynamics. Conversely, a positive correlation would imply that hosts might use a general mechanism to resist both parasite species. In this positive correlation case, directional selection for generally resistant hosts could erode genetic variation for resistance in host populations and squash Red Queen dynamics [57] – provided that this general resistance is not traded-off against another parasite-independent host trait [58]. This study found no correlation between host resistance to the two parasites (Figure 2), probably because of very different underlying resistance mechanisms involved [17], [34]. We acknowledge, however, that a more powerful design using more host genotypes could more comprehensively exclude the possibility of correlations between resistance to these two parasites. The lack of a correlation between resistance to *Metschnikowia* and *Pasteuria* suggests evolution of *D. dentifera* in response to the two parasites should proceed relatively independently. Thus, we do not expect *Metschnikowia* epidemics to amplify or dampen Red Queen dynamics between *D. dentifera* and *Pasteuria*.

In conclusion, *Pasteuria* could promote genetic diversity in multiple host species through host-parasite coevolution. *Pasteuria* exhibits strong genetic specificity and high virulence with its North American host, *D. dentifera,* just as it does with its European host, *D. magna.* Can *Pasteuria* then promote host diversity in North American lakes? We cannot yet fully answer that question with these or other data, but the *D. dentifera-Pasteuria* system in isolation certainly contains several of the essential ingredients of the Red Queen. Still, it seems likely that the larger epidemics of the more common *Metschnikowia* yeast may mean that parasite-mediated selection in *D. dentifera* is driven primarily by *Metschnikowia*, making Red Queen dynamics relatively rare. This possibility further highlights the need to consider other co-occurring parasites when exploring host-parasite coevolution.
